# The Social Connectedness Index: A large-scale dataset of social ties across geographic locations

**DOI:** 10.1016/j.dib.2026.112905

**Published:** 2026-06-02

**Authors:** Drew Johnston, Theresa Kuchler, Manas Kulkarni, Johannes Stroebel

**Affiliations:** a Independent Researcher; b NYU Stern School of Business, 44 West 4th Street, New York, NY 10012, USA

**Keywords:** Social networks, Geographic social ties, Big data, Facebook data, Subnational analysis

## Abstract

This article describes a new release of the Social Connectedness Index (SCI), a large-scale measure of social ties across geographic locations constructed from Facebook friendship links. The newly available SCI data have substantially expanded global coverage and finer geographic resolution than prior versions, and provide measures of social connectedness for national and subnational units across 178 countries. The SCI dataset is based on Facebook friendship networks from January 2026 and is freely available through the Humanitarian Data Exchange. This data descriptor details the methodology used to construct the measure, the structure of the SCI dataset, and its geographic coverage at various granularities.

Specifications Table

**Table:** 

Subject	Social Sciences
Specific subject area	Social network analysis; geographic measurement of social connectedness.
Type of data	Processed
Data collection	Data derived from Facebook platform; users active in 30 days prior to January 25, 2026 with >20 friends. Home location estimated from proprietary model using self-reported location, IP addresses, and on-platform activity patterns. Geographic aggregation at country and region (GADM, geoBoundaries, NUTS, U.S. counties/ZCTAs) levels.
Data source location	Global; derived from Facebook users worldwide
Data accessibility	Repository name: Humanitarian Data Exchange (HDX) Direct URL to data: https://data.humdata.org/dataset/social-connectedness-index
Related research article	Bailey, M., Cao, R., Kuchler, T., Stroebel, J., & Wong, A. (2018). Social Connectedness: Measurement, Determinants, and Effects. *Journal of Economic Perspectives*, 32(3), 259–280.

## 1 Value of the Data

• The Social Connectedness Index provides a freely available measure of social connectedness across geographies. By leveraging social network data, the SCI overcomes longstanding measurement challenges and enables new lines of research on the role of social networks in determining economic, social, and political outcomes.
• This updated release expands on prior versions of the SCI by offering a richer set of geographic granularities, thereby enabling analysis at finer spatial scales. It is also based on a more recent snapshot of the underlying data. Together, these improvements enhance the relevance and usefulness of the SCI for contemporary research and policy analysis.
• The dataset enables researchers to study social networks at multiple geographic levels, including country–country, region–region, and country–region pairs. This allows integration with a wide range of external data (e.g., economic indicators, migration flows, health outcomes), facilitating research in economics, sociology, geography, political science, and public health.
• The data can be easily transformed to construct aggregate measures of social connectedness at higher geographic levels using population-weighted aggregation. This allows researchers to tailor the dataset to custom geographic units or match it to alternative administrative boundaries.
• Prior versions of the SCI have been used to show that social connectedness is an important determinant of international and subnational trade flows [1]; to demonstrate that the geographic spread of COVID-19 correlates with social network structure [2]; and to document that institutional investors are more likely to invest in firms located in socially connected regions [3]. The SCI has also been used to anticipate human displacement during the war in Ukraine [4], to study the effects of social network exposure on social distancing behavior during the COVID-19 pandemic [5], and to examine the role of social connectedness in bank lending [6]. Researchers interested in using the SCI may begin by merging the SCI data with region-level outcome variables of interest (e.g., trade flows, health outcomes, economic indicators) and estimating the relationship between social connectedness and these outcomes, controlling for geographic distance and other relevant covariates.

## 2 Background

Social connections between individuals and places play a central role in shaping economic, social, and political outcomes [1,[7], [8], [9]]. Yet despite their importance, comprehensive measures of social networks have historically been difficult to construct. Survey-based approaches are limited in scale, while administrative datasets typically capture only narrow dimensions of social ties.

The Social Connectedness Index (SCI) was introduced to address some of these measurement challenges [10]. Based on data from Facebook friendship networks, the SCI quantifies the intensity of social ties across geographic areas. Since its initial release, the SCI has been widely used in academic research and policy analysis, informing studies of migration, trade, product diffusion, and access to capital, among other topics [1,[2], [3], [4],^6^,[11], [12], [13]].

This note describes a new data release via the Humanitarian Data Exchange, which provides an updated version of the Social Connectedness Index as of January 25, 2026 [21]. Relative to earlier releases, the new SCI data feature several important improvements. First, the geographic coverage has been substantially expanded to include 178 countries. Second, the data are now available at finer geographic resolutions, including geoBoundaries administrative units (version 6.0.0), the 2024 NUTS classification for European regions, and GADM version 4.1 boundaries. Third, the data are based on a more recent snapshot of the Facebook friendship network, reflecting the current structure of global social ties.

## 3 Data Description

The Social Connectedness Index (SCI) dataset is publicly available via the Humanitarian Data Exchange (HDX) repository [21]. It includes measures of social connectedness across the following geographic levels—many of which are available to researchers for the first time—allowing the study of social networks at local, national, and international scales. The dataset represents a derived and processed data product: the SCI is constructed by aggregating and transforming proprietary Facebook friendship network data. The underlying user-level data are not included in the public release; only the aggregated, differentially private SCI measures are made available. The repository is organized into multiple data files corresponding to different geographic levels and pair types:

• Country-to-country pairs

• Region-to-region pairs
  ○ GADM administrative boundaries (Levels 1 and 2, version 4.1)
  ○ geoBoundaries administrative units (Levels 1 and 2, version 6.0.0)
  ○ European NUTS regions (Levels 1, 2, and 3; 2024 classification)
United States counties and ZIP Code Tabulation Areas (ZCTAs) (2025 TIGER/Line)
• Country-to-region pairs
  ○ GADM administrative boundaries (Levels 1 and 2, version 4.1)
  ○ geoBoundaries administrative units (Levels 1 and 2, version 6.0.0)
  ○ European NUTS regions (Levels 1, 2, and 3; 2024 classification)
  ○ United States counties and ZIP Code Tabulation Areas (ZCTAs) (2025 TIGER/Line)

Across all geographic resolutions, the structure of the SCI data files is standardized. Each file contains five columns, described in Table 1.

**Table 1:** Data dictionary for the SCI dataset.

Variable	Type	Description
*user_country*	string	ISO 3166-1 alpha-2 country code identifying the country of the first location in the pair (e.g., “US”, “DE”, “BR”).
*friend_country*	string	ISO 3166-1 alpha-2 country code identifying the country of the second location in the pair.
*user_region*	string	Identifier for the geographic unit of the first location, at the level of aggregation relevant for the file. For country-level files, this is identical to user_country. For subnational files, this contains the region identifier in the relevant geographic system (e.g., a GADM region code, NUTS code, FIPS county code, or ZCTA code).
*friend_region*	string	Identifier for the geographic unit of the second location, at the same level of aggregation as user_region. For region-to-country files, this column contains the country-level identifier.
*scaled_sci*	integer	The scaled Social Connectedness Index for the pair of locations, an integer value ranging from 1 (lowest relative connectedness in the file) to 1,000,000,000 (highest relative connectedness in the file). See below for details on the scaling procedure.

The first two columns identify the countries associated with the two locations in a pair, while the latter two columns specify the corresponding geographic units at the level of aggregation relevant for the file (e.g., country, administrative region, county, or ZCTA). The final column reports the scaled value of the Social Connectedness Index for that pair of locations. There are no missing values in the released files: location pairs are included only if both geographic cells meet the minimum user threshold (see Ethics Statement).

As a concrete illustration, Table 2 shows example rows from the NUTS Level 1 region-to-region SCI file.

**Table 2:** Example rows from the NUTS Level 1 SCI file.

*user_country*	*friend_country*	*user_region*	*friend_region*	*scaled_sci*
TR	FI	TR9	FI2	83
SE	ES	SE3	ES5	5,759
ES	SE	ES5	SE3	5,759
DE	PT	DE1	PT2	7,736
DE	DE	DE1	DE7	292,897
FI	FI	FI2	FI2	1,000,000,000

## 4 Experimental Design, Materials and Methods

### 4.1 Conceptual framework

The Social Connectedness Index (SCI) measures the intensity of social connections between pairs of geographic locations using friendship links on Facebook as a proxy for real-world social ties.

Facebook friendships are undirected connections established with the mutual consent of both users, with each user able to maintain up to 5,000 friends. Because these connections typically represent real-world social relationships and require active confirmation by both parties, the resulting networks offer a meaningful proxy for offline social ties. While no single platform captures all forms of social interaction, the scale and broad geographic coverage of Facebook's user base allow the SCI to provide a uniquely comprehensive view of social connectedness across regions around the world. Facebook friendship data have therefore been widely used by researchers to study social connectedness [5,8,[14], [15], [16], [17], [18]].

The Social Connectedness Index between locations *i* and *j* is formally defined as:

$$SCI_{i,j}=\frac{Friendships_{i,j}}{Pop_{i}\times Pop_{j}},$$

where $Friendships_{i,j}$ denotes the total number of Facebook friendship links between users assigned to location *i* and users assigned to location *j*, and $Pop_{i}$ and $Pop_{j}$ denote the number of Facebook users in locations *i* and *j*, respectively. Each friendship link is weighted equally, and where i=j, a small adjustment is made to the denominator to account for the fact that users cannot form friendships with themselves.

Since the SCI is scaled within each dataset to lie between 1 and 1,000,000,000, it is best interpreted as a relative measure of friendship intensity, wherein larger SCI values indicate stronger relative social connectedness. If the SCI between location *A* and location *B* is twice as large as the SCI between location *A* and location *C*, then a randomly chosen Facebook user in location *A* is twice as likely to be friends with a randomly chosen Facebook user in location *B* than with a user in location *C*. For example, in Table 2, the fact that *scaled_sci* for DE1—DE7 is substantially larger than for DE1—PT2 indicates that a randomly chosen Facebook user in Baden-Württemberg (DE1) is considerably more likely to be friends with a randomly chosen user in Hessen (DE7) than with one in the Região Autónoma dos Açores (PT2) — in fact, 292,897/7,736 = 37.9 times more likely.

Moreover, the SCI is symmetric by construction. That is, the SCI between location *i* and location *j* is equal to the SCI between location *j* and location *i*. As a result, for any given *user_region*—*friend_region* pair, the reversed pair appears in the data with the same *scaled_sci* value. This is illustrated by rows 2 and 3 of Table 2, where the SE3—ES5 and ES5—SE3 pairs have identical *scaled_sci* values.

**Fig. 1:**
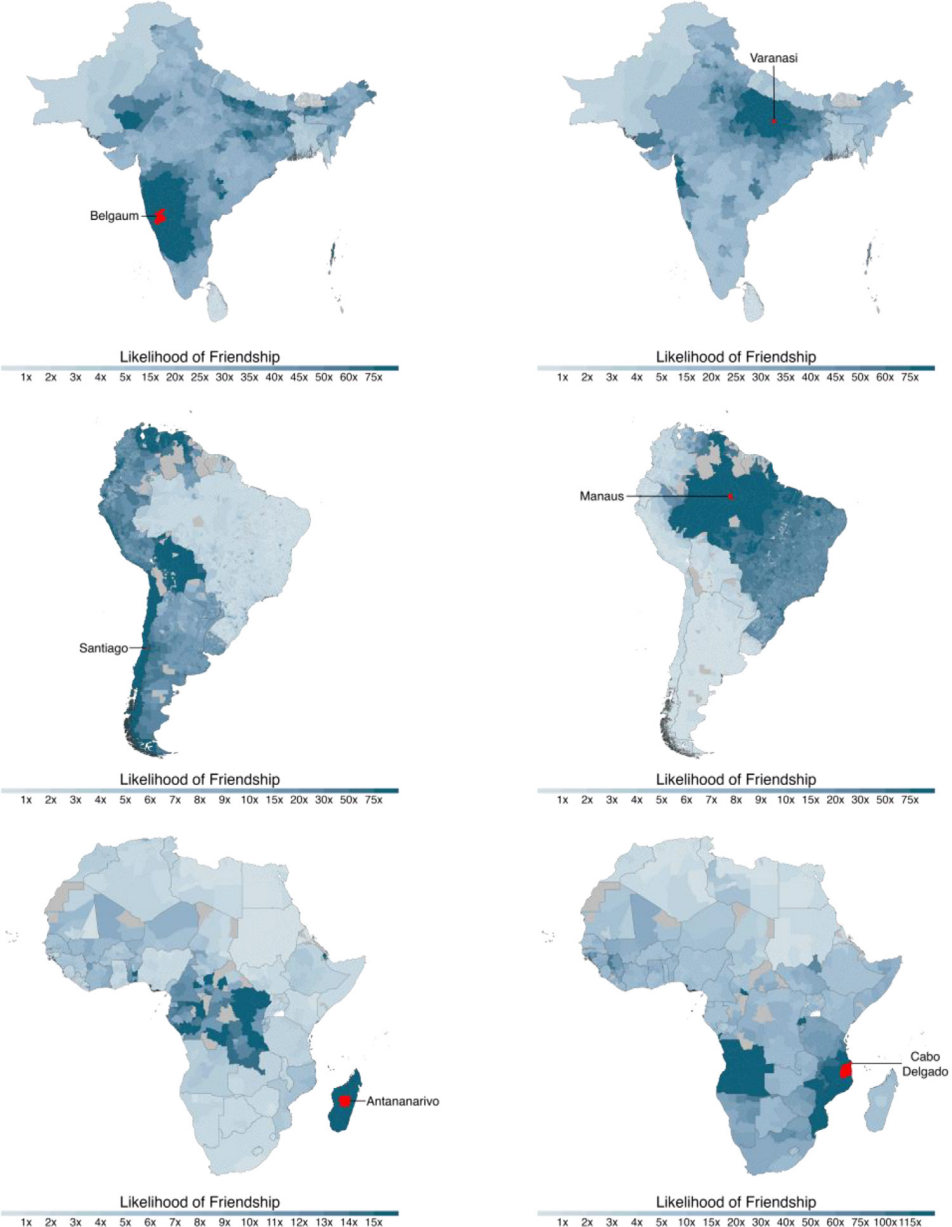
Social Connectedness in South Asia, South America, and Africa

Fig. 1(1)–(6) show the Social Connectedness Index of various regions in South Asia, South America, and Africa. Each panel shows the relative likelihood of friendship implied by the Social Connectedness Index (SCI), obtained by normalizing SCI values for the focal region by a low-connectivity reference level (the 10th percentile among regions in the countries shown). The focal region is highlighted in red.

### 4.2 Data sample

The SCI is constructed using data from Facebook, a global online social networking platform. The underlying sample consists of Facebook users from 178 countries who were active on the platform in the 30 days prior to January 25, 2026 and who had >20 Facebook friends. As context for the scale of the platform, in December 2025 Meta reported 3.58 billion daily active people across its family of apps [19].

### 4.3 Data processing pipeline

The SCI is constructed through a multi-step data processing pipeline, described below. While the pipeline is implemented on proprietary infrastructure and certain components (such as the location inference model) cannot be fully disclosed due to platform constraints, we describe each step in sufficient detail to allow researchers to understand the logic and structure of the data generation process.

Step 1: User selection and geographic assignment. We begin by selecting all Facebook users who were active on the platform within the 30 days prior to January 25, 2026 and who maintained >20 Facebook friends. Each selected user is assigned an estimated home location using a proprietary model that integrates self-reported location data, IP address information, and patterns of on-platform activity. This estimated location is then mapped to geographic identifiers across multiple supported geographic systems (country, GADM Levels 1 and 2, geoBoundaries Levels 1 and 2, NUTS Levels 1–3, U.S. counties, and U.S. ZCTAs) using a crosswalk table that links geographic coordinates to administrative boundaries. The result is a user table in which each user is associated with a country code and a set of subnational region identifiers across all supported geographic systems.
Step 2: Friendships. We construct an ego-alter friendship table by joining the Facebook friend list—which records all bilateral friendship links—to the user table from Step 1. This join is performed on both sides of each friendship link, so that each ego-alter pair is combined with the country and region identifiers for both the ego (user) and the alter (friend). The result is a table of friendship edges in which both endpoints have full geographic attributes.
Step 3: Geographic aggregation. For each configured geographic pair type (e.g., country-to-country, GADM Level 1-to-GADM Level 1, U.S. county-to-country), we aggregate friendship counts at the level of the user-region × friend-region cell pair by counting the total number of friendship links between users in each pair of geographic cells. We also compute the total number of Facebook users in each geographic cell.
Step 4: Differential privacy. Both the friendship counts and the population counts computed in Step 3 are protected using differential privacy [20]. Specifically, calibrated μ-Gaussian noise is added to the aggregate counts. This step is described in more detail in the Ethics Statement section below.
Step 5: SCI computation. Using the differentially private friendship counts and population counts, we compute the raw SCI for each pair of geographic cells as:
  $$SCI_{i,j}=\frac{DP\;Friendships_{i,j}}{DP\;Pop_{i}\times \left(DP\;Pop_{j}-\delta_{i,j}\right)},$$
  where $DP\;Friendships_{i,j}$ is the differentially private count of friendship links between cells *i* and *j*, $DP\;Pop_{i}$ and $DP\;Pop_{j}$ are the differentially private population counts for cells *i* and *j*, and $\delta_{i,j}$ equals 1 if cells i and j are identical (to account for the fact that users cannot befriend themselves) and 0 otherwise.
Step 6: Symmetrization. For symmetric geography pairs (i.e., where both sides use the same geographic system, such as county-to-county), we symmetrize the SCI matrix. Because the underlying friendship data are undirected, the raw counts are approximately symmetric, but small asymmetries can arise from the differential privacy noise. The symmetrization step ensures that $SCI_{i,j}=SCI_{j,i}$ exactly in the released data, by retaining one entry per unordered pair and duplicating it for the reverse direction.
Step 7: Scaling and export. The raw SCI values are transformed to integers in the range [1, 1,000,000,000] via min-max normalization. Specifically, for each geographic pair type, the raw SCI value is transformed as:
  $$Scaled\;SCI=\lfloor \frac{999,999,999\;\times \left(SCI_{raw}-SCI_{min}\right)}{SCI_{max}-SCI_{min}}\rfloor +1,$$
  where $SCI_{min}$ and $SCI_{max}$ are the minimum and maximum raw SCI values across all location pairs in the file, respectively. This transformation maps the lowest raw SCI to 1 and the highest to 1,000,000,000. Because the scaling is applied independently within each file, *scaled_sci* values are comparable within a given file but are not directly comparable across files corresponding to different geographic pair types or resolutions. For example, a *scaled_sci* value of 500,000 in the country-to-country file does not represent the same absolute level of connectedness as a *scaled_sci* value of 500,000 in the U.S. county-to-county file.

The resulting files are exported as CSV files. For geographic pair types with a very large number of region pairs (e.g., GADM Level 2, U.S. ZCTAs), the output is sharded into multiple files based on the user-side country or region to maintain manageable file sizes.

### 4.4 Data stability

Although the underlying Facebook data evolve continuously, the SCI measures a relatively stable underlying object: the geographic structure of real-world social networks. Prior research has shown that social connectedness measured at a single point in time predicts outcomes such as trade flows many years into the past [1], suggesting that the underlying object—how strong the social connections between different countries or regions are—does not vary at high frequency.

Consistent with this interpretation, the SCI between location pairs in the newly released data is highly correlated with the SCI in an earlier data release from 2021, with a correlation of ρ=0.97 at the country level and a correlation of ρ=0.97 at the U.S. county level. These findings also suggest that the 2026 release of the SCI should be useful to researchers for many years to come.

### 4.5 Geographic aggregation

The SCI can be aggregated to higher levels of geographic aggregation using a formula introduced by Bailey et al. [1]. Specifically, the SCI between two locations *i* and *j* is defined as the probability that a representative Facebook user in location *i* is friends with a representative Facebook user in location *j*. This measure is equivalent to a population-weighted average of the SCI across all subregions within the two locations.

For example, let R(i) denote the set of regions in country *i*, and R(j) the set of regions in country *j*. Let $Friendships_{r_{i},r_{j}}$ be the total number of Facebook friendship links between individuals in regions $r_{i}\in R\left(i\right)$ and $r_{j}\in R\left(j\right)$. Let $Pop_{r_{i}}$ and $Pop_{r_{j}}$ denote the total (Facebook) population in regions $r_{i}$ and $r_{j}$, respectively, and $PopShare_{r_{i}}$ and $PopShare_{r_{j}}$ denote the share of the population in those regions within their respective countries, such that $\sum_{r_{i}\in R\left(i\right)}PopShare_{r_{i}}=1$ and $\sum_{r_{j}\in R\left(j\right)}PopShare_{r_{j}}=1$. The SCI between countries *i* and *j* is then given by:

$$SCI_{i,j}=\sum_{r_{i}\in R\left(i\right)}\sum_{r_{j}\in R\left(j\right)}PopShare_{r_{i}}\times PopShare_{r_{j}}\times SCI_{r_{i},r_{j}}.$$

This formula provides a flexible framework for aggregating the SCI to higher geographic levels. In the absence of access to Facebook user counts for each region, population counts and shares from administrative data can be used as proxies.

### 4.6 Bias and representativeness

Although the SCI offers a uniquely large-scale measure of social connectedness, it is important for researchers to consider potential biases arising from the composition of the Facebook user base. The 178 countries in the dataset represent the vast majority of the global population. However, the share of individuals using Facebook varies across countries, raising questions about the representativeness of the SCI as a measure of overall social connectedness in areas with lower Facebook penetration.

Specifically, Facebook usage rates may vary along dimensions such as age, income, education, urban/rural residence, and internet access. For example, in countries or regions where internet access is not ubiquitous, the Facebook user base may disproportionately represent urban, younger, or higher-income populations. These patterns could introduce biases into the SCI, for example by underrepresenting social ties among older or more rural populations.

Despite these potential concerns, Facebook provides the most comprehensive global social network data currently available, with 3.58 billion daily active people across Meta's family of apps as of December 2025 [19]. The SCI has been validated in numerous prior studies, which have shown that it strongly predicts a wide range of real-world outcomes—including trade flows [1], migration patterns [7,12], the spread of communicable diseases [2], and investment decisions [3]—suggesting that the measure captures meaningful variation in the true underlying structure of social networks. We therefore construct and release data for all countries where Facebook is available, while encouraging researchers to consider the potential implications of differential Facebook adoption rates when interpreting the SCI in specific analytical contexts.

### 4.7 Replication code

We provide a public GitHub repository containing replication code for the figures in this paper, as well as sample scripts demonstrating how the SCI data can be used.

## Limitations

While the SCI provides a measure of social connectedness at unprecedented scale, it is important to recognize its limitations. First, the index only reflects social ties among Facebook users. Although Facebook usage is widespread and relatively representative along many dimensions, this representativeness might vary across locations, for example when internet access is not ubiquitous. Second, the SCI measures the prevalence of friendship links but not their intensity, frequency of interaction, or context. Two pairs of locations may exhibit similar SCI values even if the nature of the underlying social relationships differs somewhat, for example because countries might differ in the norms around adding friendship links to individuals not known in real life. Third, the application of differential privacy protections introduces a small amount of noise into the published SCI values. While this noise is small relative to the signal in the data, it may be relatively more consequential for location pairs with low underlying connectedness or for geographic cells with populations close to the minimum threshold of 500 users.

## Ethics Statement

Throughout the construction of the SCI, we implement multiple layers of safeguards to protect user privacy. First, geographic units with fewer than 500 Facebook users are excluded from the data release. This minimum cell size threshold ensures that the published SCI values are based on sufficiently large populations, reducing the risk that patterns in the data could be used to infer information about specific individuals or small groups. Second, all published statistics are protected using μ-Gaussian Differential Privacy [20]. Differential privacy provides a formal, mathematical guarantee that the inclusion or exclusion of any single individual's data has a bounded effect on the published statistics. We add μ-Gaussian noise to the aggregate counts before the SCI is computed. This means that the released SCI values are computed from noisy (differentially private) counts rather than from the exact underlying data, providing a rigorous privacy guarantee.

These safeguards ensure that the SCI dataset can be used for research purposes without compromising the privacy of individual Facebook users. No individual-level data are included in the public release; all published values are aggregated, noisy statistics computed over populations of at least 500 users.

## CRediT authorship contribution statement

**Drew Johnston:** Conceptualization, Methodology, Investigation, Writing – original draft, Writing – review & editing. **Theresa Kuchler:** Conceptualization, Methodology, Investigation, Writing – original draft, Writing – review & editing. **Manas Kulkarni:** Conceptualization, Methodology, Investigation, Writing – original draft, Writing – review & editing. **Johannes Stroebel:** Conceptualization, Methodology, Investigation, Writing – original draft, Writing – review & editing.

## Data Availability

HDXSocial Connectedness Index (Original data)
